# Effects of virtual rehabilitation versus conventional physical therapy on postural control, gait, and cognition of patients with Parkinson’s disease: study protocol for a randomized controlled feasibility trial

**DOI:** 10.1186/s40814-017-0210-3

**Published:** 2017-12-06

**Authors:** Keyte Guedes Silva, Tatiana Beline De Freitas, Flávia Doná, Fernando Freitas Ganança, Henrique Ballalai Ferraz, Camila Torriani-Pasin, José Eduardo Pompeu

**Affiliations:** 1 Department of Neuroscience and Behavior, Institute of Psychology, University of São Paulo, 1721 Professor Mello de Morais Avenue, Cidade Universitária, São Paulo, SP 05508-030 Brazil; 2 Motor Behavior Laboratory, School of Physical Education and Sports, University of São Paulo, 65 Professor Mello Moraes Avenue, Cidade Universitária, São Paulo, SP 05508-030 Brazil; 3 Anhanguera Educational, 3305 Raimundo Pereira de Magalhães Avenue, Pirituba, São Paulo, SP 05145-200 Brazil; 4 Department of Otorhinolaryngology and Head and Neck Surgery, Federal University of São Paulo, 947 Pedro de Toledo Street, Vila Clementino, São Paulo, SP 04039-002 Brazil; 5 Department of Neurology, Federal University of São Paulo, 650 Pedro de Toledo Street, Vila Clementino, São Paulo, SP 04039-002 Brazil; 6 Department of Physical Therapy, Speech and Occupational Therapy, School of Medicine, University of São Paulo, 51 Cipotânea Street, Cidade Universitária, São Paulo, SP 05360-000 Brazil

**Keywords:** Parkinson’s disease, Postural balance, Gait, Physical therapy modalities, Video games

## Abstract

**Background:**

There is an association among postural instability, gait dysfunction, and cognitive impairment in subjects with Parkinson’s disease (PD). Difficulty in dividing attention, response inhibition, and visuospatial attention deficiencies may contribute to the impairment of motor performance during daily activities. There are strong evidences that physical therapy can prevent physical and cognitive decline in individuals with PD. Recently, the European Physiotherapy Guideline (EPG) was developed based on randomized clinical trials about the effectiveness of the physical therapy to improve the functional deficiencies of individuals with PD. The EPG did not include the use of promising new intervention as virtual reality in PD due the lack of studies about its safety, feasibility and effectiveness. Therefore, this study protocol had as objective to evaluate the feasibility, safety and effectiveness of a physical therapy program-based on the European Physiotherapy Guideline (EPG) compared to Kinect-based training on postural control, gait, cognition, and quality of life (QoL) of Individuals with PD.

**Methods/design:**

A single-blind, parallel, randomized, controlled feasibility trial will be conducted with a sample of 32 individuals diagnosed with idiopathic PD. Participants will be allocated into control group (CG) and experimental group (EG). The intervention of the CG will be conventional physical therapy, and the intervention of the EG will be a supervised practice of five Kinect games. Both groups will perform 14 sessions of 1 h each one, twice a week over 7 weeks. Process outcomes will be safety, feasibility, adherence, and acceptability. Safety will be assessed by the proportion of participants who experienced intervention-related adverse events or any serious adverse event during the study period. Feasibility will be assessed through the scores of the games recorded in all training sessions. Adherence will be assessed through the participant’s attendance. Acceptability will be the motivation of the participants regarding the interventions. Clinical outcomes will be (1) postural control, (2) cognitive function, (3) balance, (4) gait, and (5) QoL. Individuals will be assessed pre- and post-interventions and after 30 days by a blinded evaluator.

**Discussion:**

This protocol will clarify if an intervention based on Kinect games will be feasible, safe, and acceptable for individuals with PD compared to conventional physical therapy. We will verify whether the proposed interventions can improve clinical outcomes as postural control, gait, cognition, and QoL of individuals with PD. Our hypothesis is that both Kinect games and conventional physical therapy will be feasible, safe, and acceptable for individuals with PD and will promote positive clinical effects. The results of this feasibility study will be used to design a future definitive clinical trial.

**Trial registration:**

Unique identification number in WHO Trial Registration: U1111-1171-0371. Brazilian Clinical Trial Registration Number RBR-27kqv5, registration date: February, 2016.

**Electronic supplementary material:**

The online version of this article (10.1186/s40814-017-0210-3) contains supplementary material, which is available to authorized users.

## Background

The impairment of gait, balance, and cognition of individuals with idiopathic Parkinson’s disease (PD) [1] increases the incidence of falls and impairs daily living activities [2]. Physical therapy can decrease the deleterious effects of PD that are resistant to dopaminergic replacement, such as festination, hesitation, freezing of gait, axial motor dysfunctions, and falls [3–6]. Based mainly on the finds of randomized clinical trials, the European Physiotherapy Guideline (EPG) for PD was developed and updated by specialists [7, 8]. There is strong evidence that physical therapy through different strategies can improve motor and cognitive functions of individuals with PD [9–13].

Practice-oriented task approach is recommended to facilitate the motor learning process and transferring for daily living activities [5–7]. The cue strategies can be used to improve gait, and specific exercises can improve balance [5, 6, 8].

Besides the well-consolidated conventional physical therapy, new therapeutic strategies based on practice-oriented task approach and visual and auditory feedback have shown positive effects on PD, among them the virtual reality training through video games [9, 10]. There are some studies suggesting that video games can promote integrated motor-cognitive training with the potential to improve balance, motor learning, cognition, and independence on daily living activities of individuals with PD [9, 11–13]. Through video games, individuals may be able to perform complex tasks (fast and large movements involving the whole body, rather than performing the movement in a single joint). Besides this, video games provide auditory and visual feedback that can contribute to physical performance [14, 15].

Amid recent technological advances, the Microsoft Kinect sensor (Kinect) is a potential low-cost alternative resource that can benefit in the treatment of individuals with PD. Pompeu et al. [16] assessed the safety and feasibility of Kinect games for individuals with PD in a pilot study with a small sample (*n* = 7) and without a control group. Due to the methodological issues of the pilot study of Pompeu et al. [16], a more robust randomized clinical feasibility trial is needed to clarify the safety and feasibility of the Kinect games compared to a conventional intervention.

The objective of this study will be to evaluate the safety, feasibility, adherence, acceptability, and clinical outcomes (postural control, gait, cognition, and QoL) of the Kinect game-based intervention compared to conventional physical therapy in individuals with PD. We hypothesize that the Kinect games will be feasible, safe, and acceptable and will provide improvements in the postural control, gait, cognition, and QoL of individuals with PD.

## Methods/design

### Trial design

This study will be a single-blind, parallel-group, randomized controlled feasibility trial. Participants will provide written informed consent prior to taking part in the study. The protocol will follow the CONSORT guidelines for reporting of non-pharmacological interventions.

The study was approved by the Ethics Committee of the Institute of Psychology, University of São Paulo, Brazil, number 1.506.842, and registered in ensaiosclinicos.gov (RBR-27kqv5). The current study was developed based on Standard Protocol Items: Recommendations for Interventional Trials (SPIRIT) (Additional file 1).

### Study setting

The study will be conducted in three different settings: (1) Motor Behavior Laboratory, School of Physical Education and Sports, University of São Paulo, Brazil; (2) Laboratory of Study and Research in Rehabilitation of Body Balance and Social Inclusion, Anhanguera Educational, São Paulo, SP, Brazil; and (3) Movement Disorders Unit, Federal University of São Paulo, São Paulo, SP, Brazil.

### Participants

#### Inclusion criteria

Individuals diagnosed with PD living in the city of São Paulo (Brazil) will be recruited. It will select individuals who meet the following inclusion criteria: 50–80 years of age, idiopathic PD diagnosed according to the criteria of the UK Brains Bank Parkinson’s Society [17], without clinical fluctuation, and in stages I to III of the modified Hoehn and Yahr [18]. All individuals should be treated with levodopa and/or their synergists. Moreover, they should be able to walk independently with or without assistive device and without signs of cognitive decline, defined according to the cutoff scores of the Mini Mental State Examination (MMSE) [19], adjusted according to the educational level (> 20 for illiterates, > 25 for individuals with 1 to 4 years of education, > 26 for individuals with 5 to 8 years, > 28 for individuals with 9 to 11 years, > 29 for individuals with more than 11 years of education) [19].

#### Exclusion criteria

It will exclude individuals with biomechanics, and significant cardiovascular or respiratory alterations that could compromise training performance. All individuals should be in medical treatment, and any clinical fluctuation reported at their medical records will be excluded.

### Procedure

**Fig. 1:**
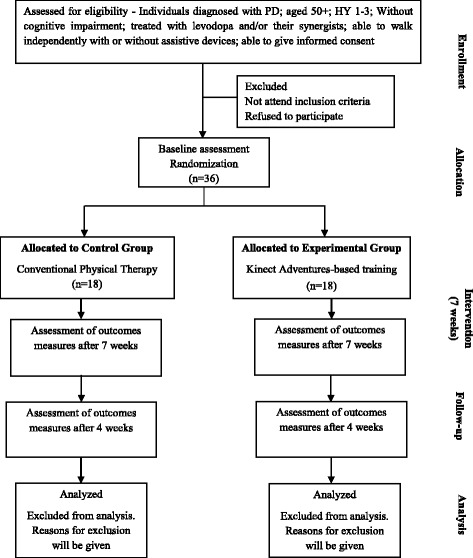
Flow diagram of trial design. Abbreviations: PD Parkinson’s disease, HY Hoehn and Yahr, MMSE Mini Mental State Examination, n number of participants

Figure 1 outlines the study phases. Participants in both groups will be allocated to an intervention at the ratio of 1:1. The trial will be carried out over a 7-week period.

### Outcome measures

Individuals will be evaluated during the “on” period by two blinded, trained physical therapists. The evaluators will be blinded to the type of intervention that individuals will receive. Individuals will be oriented to not comment to the evaluators about any details of the intervention that they will receive. In compliance with the pharmacodynamics of levodopa (onset is 20 to 40 min and duration of effect is 2 to 4 h after medication), individuals will be evaluated in the period of 2 h after medication, according to Lipp et al. [20]. This is the time at which the individual had better engine performance related to the effect of levodopa.

Table 1 details the outcome measures that will be used to assess the treatment effects. All measurements will be taken at baseline (week 0), at the end of the intervention (week 7), and 30 days after the second evaluation (follow-up). For a month after the intervention, the individuals will be instructed to not begin any new physical activity in order to not interfere in the evaluation of the long-lasting effects of the intervention.

**Table 1:** Summary of the outcome measures to be used in the study design

Outcome	Measurement tool
Primary outcomes	
Center of pressure and limits of stability	Posturography through the Balance Rehabilitation Unit
Secondary outcomes	
Postural control	Mini Balance Evaluation Systems Test
Functional mobility	Dynamic Gait Index and Functional Gait Assessment
Physical functioning	Five Times Sit-to-Stand Test and 10-Meter Walk Test
Cognition	Montreal Cognitive Scale
Quality of life	Brazilian version of the Parkinson’s Disease Questionnaire

Scales that will be used in the study protocol

#### Process outcomes

Process outcomes will be safety, feasibility, adherence, and acceptability. Safety will be assessed by the proportion of participants who experienced intervention-related adverse events or any serious adverse event during the study period. An adverse event was defined as any untoward medical occurrence, such as falls, joint and muscular pain, syncope, dizziness, or any other medical condition that requires hospitalization or leads to disability.

Feasibility will be assessed through the scores of the games recorded in all training sessions, according to Pompeu et al. [16]. An increase in scores will indicate that the participant is able to not only play but also improve his performance in the games. To determine the adherence, we will collect each participant’s attendance records before and after the training sessions. We will conclude that the exercise program is feasible if we obtain > 50% attendance of all sessions per patient and a mean of attendance > 80% per session.

The acceptability will be evaluated through a questionnaire developed by the researchers of this study in which the individuals will answer questions related to their motivation to be involved in the intervention.

#### Clinical outcomes

Static postural control and limits of stability (LOS) will be assessed using the AMTI Accugait Optimized™ force platform (AMTI, Inc., Newton, MA). The Accugait measures the three-dimensional applied forces (Fx, Fy, Fz) and moments (Mx, My, Mz) involved in balance and uses established algorithms to compute the location of the center of pressure (COP) and its associated variables with acquisition frequency set to 100 Hz. Data will be acquired, recorded, and analyzed using Balance Clinic software (balance software for AMTI’s Accusway plus balance platform, version 2.02.01).

The measures will be the area of a 95% confidence ellipse of the COP excursion in the anterior-posterior and mediolateral directions and the mean velocity of the COP in two trials of 60 s, under six conditions: (1) orthostatic position on firm ground with open eyes (EO); (2) orthostatic position on firm ground with closed eyes (EC); (3) dual-task (DT): orthostatic position on firm ground with OE and performing two fluency tasks and counting backwards from 150 by subtracting 3’s (trial 1) and counting backwards from 180 by subtracting 3’s (trial 2); (4) orthostatic position on firm ground performing excursion in the anterior-posterior and mediolateral directions within the limit of stability; (5) orthostatic position on a foam pillow with OE; and (6) orthostatic position on a foam pillow with EC.

Postural control will be evaluated by the Mini Balance Evaluation Systems Test (Mini-BESTest), a scale that consists of 14 items that focus on dynamic balance, comprising four areas: anticipatory postural adjustments, postural responses, sensory orientation, and balance during walking. Each item is scored from zero to two; zero indicates that a person is unable to perform the task, while two means that the performance is normal. The maximum score is 28 [21].

Functional mobility will be evaluated by the Dynamic Gait Index (DGI), which measures dynamic balance, mobility, activities of daily living, and the risk of falls. This scale consists of eight tasks involving gait in different sensorial contexts, including level surfaces, changes in gait speed, horizontal and vertical movements of the head, walking over and around obstacles, turning on their own body axis, and up and down stairs. Each item is scored from zero to three; zero indicates gait with severe impairment, while three indicates normal gait. The maximum score is 24 points, and a score of 19 or less predicts risk for falls [22].

Gait will be assessed by Functional Gait Assessment (FGA), which consists of 10 items, seven of which include items that comprise the DGI, with additional tests: posterior gait, gait on the basis of reduced support, and gait with closed eyes. Each item is scored from zero to three, in which a score of zero indicates an inability to perform the task, while three is normal. According to the author, the best score is the maximum of 30 [23].

The analysis of the dual task will be evaluated by the Five Times Sit-to-Stand Test (SST) and 10-Meter Walk Test (10WT). The SST will be performed according to the description of Duncan, Leddy, and Earhart [24]. The individual will sit in the center chair, with back straight, feet parallel, and arms folded across the chest; they will be asked to get up and sit straight as soon as possible five times. A verbal command will be given to start and end the test, and during the test, they will be not given any form of incentive.

The 10WT will be realized in single and dual tasks. Participants will cross a distance of 10 m as described by Novaes, Miranda, and Dourado [25]. To eliminate the acceleration and deceleration components, the participants will be instructed to begin walking 1.2 m before the beginning of the course and to finish 1.2 m after the end of the course at usual speed. For both the SST and 10WM, in the single-task condition, it will record the time in seconds to complete the route. In the dual-task condition, it will measure the task execution time (in seconds) and the number of words spoken by the patients during the test.

Cognition will be evaluated by the Montreal Cognitive Scale (MoCA). The instrument assesses different cognitive domains, such as visuospatial and executive functions, namely, memory, attention, language, abstraction, delayed recall, and guidance. The total score of the scale is 30, and scores equal to or higher than 26 indicate normal performance [26].

Quality of life will be assessed by the Brazilian version of the Parkinson’s Disease Questionnaire (PDQ-39). It comprises 39 items divided into eight dimensions: mobility, activities of daily living, emotional well-being, stigma, social support, cognition, communication, and body of discomfort. Each item is scored from zero to four. The overall score ranges from zero (no problem) to 100 (maximum problem), but lower scores indicate better perception by the individual of their quality of life [27].

To evaluate the performance game, the score for each game will be compared in each session, comparing the first session (pretest) with the last session (posttest) and retention (30 days after).

### Interventions

The individuals of both groups will participate in 14 training sessions (60 min, two times per week), during the “on” phase of dopaminergic medication.

The interventions were designed in order to guarantee the same intensity and quantity of exercises for both groups. The intensity of both group interventions will be adjusted to achieve moderate intensity. We hypothesize that both interventions will motivate the participants in different ways. As conventional physical therapy will be performed in groups, the social aspect will probably be strong in this group, while the novelty of the technology and the amusing nature of the interactive video games may motivate the participants of this group.

#### EPG-based training (control group)

The intervention of the control group (CG) will be developed in accordance with the practice recommendations in the guidelines for rehabilitation in Parkinson’s disease [8]. Sessions will be conducted by a trained physical therapist with a maximum of 20 participants and an instructor-to-participant ratio of 1:2.

The protocol will be structured in order to stimulate (1) muscle flexibility, (2) muscle strength, (3) static and dynamic balance, (4) physical fitness, and (5) transfer through cognitive movement strategies (see Table 2 for details).

**Table 2:** Control group exercise intervention description

Modality	Format	Time	Exercises	Intensity	Examples of exercises
Warm up	Group	5 min	Anterior-posterior gait Lateral gait	N/A	Gait training in association with active movements of the upper limbs Dual task training associating cognitive and motor activities
Balance	Group	15 min	Stable and unstable surfaces Progressive levels of complexities in reducing the base of support With visual and auditory cues	N/A	Tandem position Standing on one leg Raising body on the forefoot Without visual afferent (open and closed eyes)
Physical conditioning	Free rhythm set to music	15 min	Gait with change in speed Gait bypassing and overcoming obstacles Circuit	Intensity of the exercises; will use the Borg scale and heart rate	Up and down stairs Multiple direction changes “Zig-zag” movements Walk in tandem
Strength	Individual training	10 min	Concentric isotonic exercises in the sitting or supine position	Three sets of 10 repetitions with elastic bands or weights on the ankles 60 s intervals	Trunk extensors, scapular stabilizers, knee extensors, and hip abductors and extensors
Postural transfers training	Group	5 min	Movement fragmentation conducting the training activities approaching the day-to-day	Requested each subsequent session to repeat the trained movement	Get up and sit down in the chair Get up and lie in bed Get up from the floor
Flexibility and cooldown	Group	5 min	Static muscle stretching in sitting or supine position	3 RPE 30 s	Cervical and trunk extensors, shoulder flexors, elbow extensors, and flexors of the hip and knee

N/A not applicable, RPE repetitions

A comprehensive exercise program will be designed, with a focus on multiple systems involved in postural control, such as postural reactions, sensory integration, and biomechanical constraints. This program will also include gait training in dual tasks and emphasizing of the swing phase, as well as practice for fall-prone function.

The sessions will begin with 5 min of warming exercises in order to prepare individuals for the next exercises. The static and dynamic balance training will be taught for 15 min to perform specific exercises that challenge balance.

Physical conditioning will be conducted for 15 min. In order to assess the intensity of the exercises, we will use the Borg scale [28], which measures the effort level of the subject, as well as heart rate. Both measurements will be used before and after aerobic training. Muscle strength training will be performed for 15 min and will include progressive weight training involving the major muscle groups.

Training postural transfers will be performed for 10 min through specific daily activities that hinder the independence of individuals with PD. Finally, in the last 5 min, flexibility training will be performed in order to relax the muscles and cool down.

#### Kinect adventure-based training (experimental group)

The experimental group (EG) will practice, individually and randomly, four games using the Kinect Adventure program for Xbox 360, developed by Microsoft. The selection of games was done based on a previous pilot study [16].

The individuals will practice five attempt games: (1) *20,000 Leaks*, (2) *Space Pop*, (3) *Reflex Ridge*, and (4) *River Rush*. Table 3 shows the training schedule for the games, together with their motor and cognitive demands. The trained physical therapists that will supervise the practice with the games will use the same verbal instructions during the sessions.

**Table 3:** Experimental group exercise intervention description

Game training	Main motor demands	Main cognitive demands
20,000 Leaks	Constant displacement of the individual’s center of mass through movement of the upper limbs and neck Crouching Jumps Multidirectional steps	Fast reaction time Immediate planning and execution Visuospatial attention Dual task
Space Pop	Multidirectional steps Transference of weight between lower limbs Constant displacement of the individual’s center of mass Movement of upper limbs	Immediate planning and execution Visuospatial attention Fast reaction time Dual task
Reflex Ridge	Crouching Jumps Lateral displacement of the center of mass Side steps	Fast reaction time Immediate planning and execution Decision-making Shifting of attention
River Rush	Lateral displacement of the center of mass Crouching Jumps Side steps	Fast reaction time Immediate planning and execution Decision-making Shifting of attention

Adapted from Mendes et al. (2015, p.71) [32]

On the first day of training, there will be a demonstration of each game for the subject. Thereafter, individuals will have two attempts to familiarize themselves with the game and will receive the examiner’s instructions in order to correctly perform the movement to achieve the goal of the game.

After the familiarization phase, individuals will perform the training of the games for 14 sessions, without the interference from the physical therapist. The scores on the games will be recorded in each session of training. After 30 days of training, individuals will perform retention test that will consist of performing another block with five attempts for each game.

Below is the explanation of the steps of the research contained in Table 4, showing the content for the schedule of enrollment, interventions, and assessments.

**Table 4:** Content for the schedule of enrollment, interventions, and assessments

	Study period				
Time point	Day 1	Day 2	Days 3 to 16	Day 17	Day 47
Enrollment	X				
Eligibility screen	X				
Informed consent	X				
Allocation	X				
Interventions			X		
EG			X		
CG			X		
Assessments		X		X	X
Initial assessment (Pre)		X			
Final assessment (Post)				X	
Follow-up assessment					X

*EG* experimental group (Kinect adventure-based training), *CG* control group (European Physiotherapy Guideline-based Training)

### Adverse effects of the intervention

Adverse events are defined as any sign of pain perceived by the participants due to the training protocol lasting for 2 days or more. In addition, the occurrence of fall or injury to other body parts during training sessions will be considered as adverse effects. Falls are defined as “an unexpected event in which the participants come to rest on the ground, floor, or lower level” [29].

### Sample size

No formal sample size calculation will be performed for this feasibility study. We will follow the sample size recommendations for pilot randomized controlled trials [30], and we aimed to recruit 18 participants for each group (i.e., total sample size of 36) considering a dropout of 20%. This number of participants is deemed adequate to provide sufficient information on key feasibility issues such as recruitment and acceptability of the intervention.

### Randomization, allocation concealment, and blinding

The participants who fulfill the inclusion criteria will be randomly assigned to one of two groups (experimental or control group) with the same number of participants. Stratified randomization will be achieved by generating a separate block for each stage of PD stages (I, II and III). After all, individuals have been identified and assigned into the blocks, simple randomization will be performed within each block to assign individuals to one of the groups (EG or CG) [31]. Two physical therapists blinded to treatment allocation will record all measurements. The evaluators will be blinded to the type of intervention that individuals will receive. Individuals will be oriented to not comment to the evaluators about any details of the intervention that they will receive.

### Statistical analysis

The data analysis for the randomized controlled feasibility trial will be undertaken on an intention-to-treat basis. Demographic data will be presented to evaluate baseline comparability of the groups. Descriptive statistics will be used to characterize the groups at baseline and to present feasibility outcomes. Comparisons of the clinical outcomes between CG and EG will be performed to investigate the feasibility of this trial and to calculate estimates for likely effect sizes and 95% confidence intervals. Differences between the two groups will be presented as an unadjusted mean difference for continuous variables with their associated 95% confidence intervals; effect size will be calculated for paired *t*-test and *p* value with an alpha of 0.05, power of 0.8, and 0.5 correlations between baseline, at the end of the trial, and at follow-up. At the end of the study, data collection and statistical code will be publicly available, while maintaining the anonymity of the participants. All data will be analyzed using SPSS software version 22 (SPSS, Chicago, Illinois).

## Discussion

This protocol will clarify whether an intervention based on Kinect games will be feasible, safe, and acceptable for individuals with PD compared to conventional physical therapy. We will verify whether the proposed interventions can improve clinical outcomes as postural control, gait, cognition, and QoL of individuals with PD. Our hypothesis is that both Kinect games and conventional physical therapy will be feasible, safe, and acceptable for individuals with PD and will promote positive clinical effects. The results of this feasibility study will be used to design a future definitive clinical trial.

There is strong evidence of the effectiveness of conventional (or usual) physical therapy to improve the motor and non-motor functions in individuals with PD [9, 12, 13]. However, to our knowledge, this will be the first randomized clinical feasibility trial that will investigate the safety, feasibility, and clinical outcomes of the Kinect games compared to conventional physical therapy in individuals with PD.

Previous studies have shown that interactive video games can improve motor and cognitive functions of individuals with PD [11, 12, 16]. Video games have emerged as a new low-cost virtual reality tool that can be potentially used in rehabilitation. The games can stimulate multi-directional displacements, weight transfer, controlled movements close to stability limits, a high number of repetitions, auditory and visual feedback, attention, planning, decision-making, sustained concentration, promoting motivation, and commitment in the tasks performed [11, 16]. However, commercial video games have not been developed specifically for individuals with neurological disorders, which could hinder their safety and feasibility to this population.

This study protocol is an innovative clinical feasibility trial regarding the evaluation of the feasibility, adherence, safety, acceptability, and effectiveness of the use of the Kinect games in comparison to conventional physical therapy in individuals with PD. The final results will be used to calculate the sample size needed for a potential larger multicenter trial. Our study may contribute to the understanding of the potential benefits of the interactive Kinect video game technology in rehabilitation of individuals with PD.

## Additional files

Additional file 1: SPIRIT Checklist. Recommended items to address in a clinical trial protocol and related documents. (DOC 120 kb)

Additional file 2: Informed consent form. (DOCX 21 kb)

## Abbreviations

10WT: 10-Meter Walk Test
BRU: Balance Rehabilitation Unit
CG: Control group
COP: Center of pressure
DGI: Dynamic Gait Index
EG: Experimental group
EPG: European Physiotherapy Guideline
FGA: Functional Gait Assessment
Kinect: Microsoft Kinect sensor
LOS: Limits of stability
Mini-BESTest: Mini Balance Evaluation Systems Test
MMSE: Mini Mental State Examination
MoCA: Montreal Cognitive Scale
PD: Parkinson’s disease
PDQ-39: Parkinson’s Disease Questionnaire
QoL: Quality of life
SST: Five Times Sit-to-Stand Test
