# Supplementary material for: Effects of virtual rehabilitation versus conventional physical therapy on postural control, gait, and cognition of patients with Parkinson’s disease: study protocol for a randomized controlled feasibility trial
Source: Pilot Feasibility Stud. 2017 Dec 6;3:68. doi: 10.1186/s40814-017-0210-3 (PMC5719545; doi:10.1186/s40814-017-0210-3)
Supplement: Supplementary file 2 — Informed consent form. (DOCX 21 kb) [file 40814_2017_210_MOESM2_ESM.docx]

**INFORMED CONSENT FORM**

**I - SUBJECT IDENTIFICATION DATA OR LEGAL RESPONSIBLE**

**1.**Name: ..........................................................................................................................................

Identity document Nº: …………………… Gender: M □ F □ Date birth: ........ / ........ / ......

Address:........................................................................................................................................ City:....................................... Zip code:...................... Telephone: DDD ....................................

**2.** Legal responsible: ...................................................................................................................

Degree of kinship, tutor, healer: ..................................................................................................

Identity document Nº: …………………… Gender: M □ F □ Date birth: ........ / ........ / ......

Address:........................................................................................................................................ City:....................................... Zip code:...................... Telephone: DDD ....................................

___________________________________________________________________________

**II - RESEARCH DATA**

**1.**Title of the research protocol:

**"Effects of Kinect Adventures on postural control, gait, cognition and quality of life of patients with Parkinson's disease: a randomized controlled trial"**

**2.** Principal Investigator: José Eduardo Pompeu

Post / occupation: Teacher / Researcher

Regional physiotherapy council Nº: CREFITO-3: 19445-F

HCFMUSP Unit: Institute of Psychology

**3.** Evaluation of the risk of research:

Minimum ■ medium □ Low □ Higher □

**4.** Duration of the research: 02 years

**III - STUDY DESCRIPTION:**

This information is being provided for their voluntary participation in this study, which aims to compare the effects of two types of training on their balance, gait, attention and quality of life. The trainings will be through the Kinect games or conventional physiotherapeutic training. The practice of this videogame or conventional physiotherapy has characteristics that can improve its balance, gait, attention and quality of life and this will be verified in this study.

If you agree to participate in this study, you will undergo an assessment of your balance, gait, attention and quality of life through tests and questions asked by two physiotherapists. This evaluation will be repeated at the end of the training by the same physiotherapists, and also, after one month of the final training. After the evaluation, it will be drawn the kind of training you will realize.

If you are drawn for training with the Kinect, you will do the individual training of four games of the videogame. But if you are randomly selected for the conventional treatment you will perform group physical activities. In both training sessions, you will be assisted by physiotherapists during exercise. Training sessions of one hour will be held twice a week during seven weeks. As balance exercises will be performed in both training sessions, you may experience imbalances, but will be supported by the researcher or instructors at all times.

In any of the treatments that you are allocated, the possibility of risks is minimal, may feel discomfort due to muscle fatigue minimum, for example. You will not have any risk of falls, because you will always be supported by the physiotherapist or a monitor. To any symptom or inconvenience that you present, request that you report to the physiotherapist or monitor that will provide you with the necessary support. If you need medical attention, the SAMU service will be activated and sent to public hospital treatment.

Although the aim of this study is to improve the balance, gait, attention and quality of life of individuals with Parkinson's disease, as this is an experimental study it is not possible to ensure that you notice any of these improvements.

You will be left with a copy of this document in case you have any doubts you may consult. But we are also willing to answer any questions or clarify any questions about the study.

The principal investigator is Professor José Eduardo Pompeu, who can be found at the address Cipotânea, 51, São Paulo – SP or on the phone 3091-8424. If you have any questions or concerns about research ethics, please contact the Research Ethics Committee of the Institute of Psychology of the University of São Paulo (CEPH-IPUSP): Professor Mello Morais, 1721 – Block G, 2° Floor, Room 27 – Zip code: 05508-030 - Phone: (11) 30914182 E-mail: cep.ip@usp.br.

You are free to withdraw your consent at any time and to stop participating in the study. Your identification and your data will be confidential, being known only by those responsible for the study. All information about the progress of the study will be passed on to you, even if they are not favorable. There will be no personal expenses for you and there will also be no financial compensation related to your participation.

I declare that, after being adequately clarified by the researcher and having understood what was explained to me, I agree to participate in the present research project.

São Paulo, _____/_____/_____

-------------------------------------- -------------------------------------

Signature of the participant José Eduardo Pompeu

or legal representative (Principal investigator)
